# American Gynecological Society

**Published:** 1890-10

**Authors:** 


### AMERICAN GYNECOLOGICAL SOCIETY.

The fifteenth annual meeting of the American Gynecological Society was held in the lecture room of the Buffalo Library, September 16th, 17th, and 18th. The roll-call showed twenty-two Fellows present. The local profession was well represented at all the meetings of the Society.

The session was opened by an address of welcome, delivered by Dr. Roswell Park, of Buffalo, after which the remainder of the morning was devoted to the discussion of the Diagnosis, Pathology and Treatment of Extra-uterine Pregnancy. The first paper on this subject was read by Dr. A. W. Johnstone, of Dansville, Ky. Dr. Johnstone advised laparatomy under all circumstances. A paper by Dr. Mann, of Buffalo, followed, in which the writer urged the trial of electricity in certain cases. Dr. J. M. Baldy, of Philadelphia, was unable to be present, and his paper was read by the secretary, as was also a paper by Dr. Hanks on the same subject. At 1 P. M. the Society adjourned and accepted an invitation to lunch at Dr. Mann's. The members of the Buffalo Medical Club and a few other Buffalo physicians were invited to meet the Fellows.

The afternoon was devoted to the reading and discussion of a paper by Dr. W. C. Ford, of Utica, entitled, The Question of Amperage in the Treatment of Fibroid Tumors by Electricity.

In the evening, members of the Society were entertained at dinner by Drs. Mann, Park, Cary and Tremaine.

The morning session of the second day was opened by a paper on Vaginal Fixation of the Stump in Abdominal Hysterectomy, by Dr. Henry T. Byford, of Chicago. The Society then listened to an address on the Sanctity of Marriage, delivered by the President, Dr. John P. Reynolds, of Boston. The morning session closed with a paper on Injuries to the Uterus during Labor, by Dr. A. J. C. Skene, of Brooklyn. The Society then adjourned to the Buffalo Club, where they were entertained at luncheon by Dr. Ford, of Utica.

In the afternoon, Dr. H. A. Kelly, of Baltimore, read a paper on Cephalematoma, and Dr. Ashby, of Baltimore, discussed the subject of Drainage after Laparatomy.

In the evening, at a business meeting, the officers elected for the ensuing year were:

President, Dr. Reeve Jackson, of Chicago.

Vice-President, 1st, Dr. J. Taber Johnson, Washington.

Vice-President, 2d, Dr. S. D. Sinclair, Boston.

Secretary, Dr. H. C. Coe, New York.

Treasurer, Dr. M. D. Mann, Buffalo.

The papers read upon the third day were as follows:

A Modification of Tait's Operation for Laceration of the Perineum through the Sphincter, by Dr. H. T. Hanks, of New York.

The Resemblance of Benign to Malignant Disease, by Dr. Edward W. Jenks, of Detroit.

Laparatomy for Intra-Pelvic Pain of Sixteen Years' Standing, by Dr. Ashby, of Baltimore.

In conclusion, it may be said that the meeting, although small, was eminently successful. The papers were all well discussed, and many new and interesting facts brought out.

After the final adjournment, the Society were the guests, at lunch, of Dr. Sutton, of Pittsburg, and then proceeded in a special car to Niagara Falls. After having visited all the points of interest at the Falls, the Society returned in time to dine at the Buffalo Club. Both the excursion to Niagara and the dinner in Buffalo were at the invitation of the Medical Club of this city.

We sincerely hope that some of our other National Medical Societies will soon choose Buffalo as their meeting-place. We can assure them of a hearty welcome from the profession here.

